# Long-Term Exposure of Early-Transformed Human Mammary Cells to Low Doses of Benzo[a]pyrene and/or Bisphenol A Enhances Their Cancerous Phenotype via an AhR/GPR30 Interplay

**DOI:** 10.3389/fonc.2020.00712

**Published:** 2020-05-29

**Authors:** Caterina F. Donini, Myriam El Helou, Anne Wierinckx, Balázs Győrffy, Sophie Aires, Aurélie Escande, Séverine Croze, Philippe Clezardin, Joël Lachuer, Mona Diab-Assaf, Sandra E. Ghayad, Béatrice Fervers, Vincent Cavaillès, Véronique Maguer-Satta, Pascale A. Cohen

**Affiliations:** ^1^Université Lyon 1, Lyon, France; ^2^CRCL-Centre de Recherche en Cancérologie de Lyon-Inserm U1052-CNRS U5286, Lyon, France; ^3^Département Cancer et Environnement, Centre Léon Bérard, Lyon, France; ^4^Faculty of sciences II, Lebanese University, Fanar, Lebanon; ^5^ProfileXpert, SFR-Est, CNRS UMR-S3453, INSERM US7, Lyon, France; ^6^Department of Bioinformatics, Semmelweis University and TTK Lendület Cancer Biomarker Research Group, Budapest, Hungary; ^7^UMR5569 (UM IRD CNRS), Montpellier, France; ^8^INSERM, UMR1033 LYOS, Lyon, France; ^9^IRCM - Institut de Recherche en Cancérologie de Montpellier, INSERM U1194, Université de Montpellier, Institut régional du Cancer de Montpellier, CNRS, Montpellier, France

**Keywords:** environmental factors, Benzo[a]pyrene, Bisphenol-A, breast cancer, tumor progression, AhR, GPR30

## Abstract

It is of utmost importance to decipher the role of chronic exposure to low doses of environmental carcinogens on breast cancer progression. The early-transformed triple-negative human mammary MCF10AT1 cells were chronically (60 days) exposed to low doses (10^−10^ M) of Benzo[a]pyrene (B[a]P), a genotoxic agent, and/or Bisphenol A (BPA), an endocrine disruptor. Our study revealed that exposed MCF10AT1 cells developed, in a time-dependent manner, an acquired phenotype characterized by an increase in cancerous properties (anchorage independent growth and stem-like phenotype). Co-exposure of MCF10AT1 cells to B[a]P and BPA led to a significantly greater aggressive phenotype compared to B[a]P or BPA alone. This study provided new insights into the existence of a functional interplay between the aryl hydrocarbon receptor (AhR) and the G protein-coupled receptor 30 (GPR30) by which chronic and low-dose exposure of B[a]P and/or BPA fosters the progression of MCF10AT1 cells into a more aggressive substage. Experiments using AhR or GPR30 antagonists, siRNA strategies, and RNAseq analysis led us to propose a model in which AhR signaling plays a “driver role” in the AhR/GPR30 cross-talk in mediating long-term and low-dose exposure of B[a]P and/or BPA. Retrospective analysis of two independent breast cancer cohorts revealed that the *AhR/GPR30* mRNA expression signature resulted in poor breast cancer prognosis, in particular in the ER-negative and the triple-negative subtypes. Finally, the study identified targeting AhR and/or GPR30 with specific antagonists as a strategy capable of inhibiting carcinogenesis associated with chronic exposure to low doses of B[a]P and BPA in MCF10AT1 cells. Altogether, our results indicate that the engagement of both AhR and GPR30 functions, in particular in an ER-negative/triple-negative context of breast cells, favors tumor progression and leads to poor prognosis.

## Introduction

Progression of human breast epithelial cells from non-cancerous to pre-malignant and of early-transformed mammary cells to malignant stages is a multiyear, multistep, multiscale, and multipath disease process. More than 85% of breast cancers are sporadic and potentially attributable to long-term exposure to environmental factors, such as chemical carcinogens (1–5). Given the increasing evidence that common environmental carcinogens play a significant role in breast cancer, increased attention has been paid to molecular mechanisms through which pollutants affect breast tumor formation, progression, and/or invasion (6–8). The identification of such molecular mechanisms could have several societal and environmental consequences, and may lead to the discovery of human biomarkers of exposure to environmental carcinogens exploitable for breast cancer prevention.

Previous *in vitro* investigations have mainly been conducted on human mammary epithelial cells or on human breast cancer cells, reflecting the impact of environmental factors on the earlier and later stages of carcinogenesis (9–13). However, little is known on the impact of exposure to pollutants on the breast early-transformed stage. Short-term exposure of cells to carcinogens at micro- to millimolar concentrations was previously typically investigated (1, 2, 14–16) which, while informative, is not optimal in mimicking natural chronic exposure to low doses of environmental carcinogens and to reflect physiologically-achievable levels of environmental mammary carcinogens. Additionally, few studies have attempted to mimic natural environmental exposure by assessing the impact of exposure to a combination of several pollutants with distinct mechanisms of action that may interact or induce a greater adverse effect than the use of individual compounds.

Benzo[a]pyrene (B[a]P), a family member of poly-cyclic aromatic hydrocarbons, is considered to be a tobacco, environmental, and dietary chemical carcinogen classified as Group 1 carcinogen by the IARC (17). B[a]P is a tumor initiator that binds and forms a complex with the aryl hydrocarbon receptor (AhR) (18–20). Upon such activation, the AhR-transcriptional complex activates specific DNA-recognition elements, such as xenobiotic response elements (XREs), and upregulates the expression of genes such as cytochrome P450 isoforms (including *CYP1A1*). These latter are involved in the metabolic activation of B[a]P in genotoxic metabolites forming DNA adducts relevant for carcinogenesis [for review, (21, 22)]. A growing body of evidence is accumulating implicating the B[a]P/AhR/CYP1A1 pathway in carcinogenesis (23–25). At early stages of carcinogenesis, short-term and millimolar B[a]P doses were shown to induce aggressiveness and transformation of non-cancerous human mammary epithelial cells (26–28). The impact of chronic and low-dose exposure of these cells to B[a]P was scarcely investigated, but seems to foster progression toward the early-transformed stage by favoring increased mesenchymal, stem-like, and anchorage-independent growth properties (9–13, 29).

Bisphenol-A (BPA) is a monomer of polycarbonate plastics and human exposure to BPA mainly occurs through the oral route due to the leaching of BPA in food and beverage containers, but non-dietary sources such as dust, air and cosmetics are also relevant (30). BPA has been the focus of widespread concern due to the fact that it interferes with endocrine signaling pathways even at extremely low doses, and thus belongs to the endocrine-disrupting compounds (EDC) (31, 32). BPA is known to bind to estrogen receptors alpha and beta (ERα and ERβ), to the G protein-coupled receptor 30 (GPR30) but also to the pregnane X receptor (PXR) (31, 33). Although several *in vivo* studies reported a carcinogenic potential of BPA [reviewed in (32)], the World Health Organization (WHO) indicated that there is currently insufficient evidence on which to base this carcinogenic potential (34). *In vitro* studies have however revealed that BPA causes adverse effects in non-cancerous mammary epithelial cells or in breast cancer cell lines, including increased cell proliferation, cell stemness, oxidative stress, and alterations of cell signaling pathways involved in carcinogenesis (13, 29, 35–38).

The MCF10 unique model of breast cancer progression comprises a series of isogenic triple-negative cell lines derived from MCF10A cells (MCF10A, MCF10AT1 and MCF10CA1a.cl1 cells). The parental cell line (MCF10A) having been originally isolated from a woman with fibrocystic change (39), the members of the MCF10 series belong to the triple negative/basal-like subtype (ER-negative, progesterone receptor (PR)-negative, HER2-negative) (40–42). These cell lines thus recapitulate the stages of mammary carcinogenesis (43), making this a valuable *in vitro* model for studying the progression of triple-negative breast cancer (44–46). In the present study, we used MCF10AT1 breast cells which represent the transformed early stage in the MCF10 unique model of breast cancer progression (43, 44) to further characterize the carcinogenic potential of B[a]P and BPA. To our knowledge, these cells have never been used to test the impact of chronic and low-dose exposure to environmental pollutants.

The main objectives of this work were to newly investigate: (i) whether long-term and low-dose exposure to B[a]P and/or BPA triggers the progression of early-transformed mammary cells to a more aggressive stage; (ii) whether their combination enhances the effect of each compound tested individually, in particular whether BPA facilitates the pro-carcinogenic activity of B[a]P; and (iii) to identify candidate strategies capable of inhibiting mammary carcinogenesis linked to chronic exposure to the environmental pollutants B[a]P and/or BPA.

Our data reveal that long-term and low-dose exposure to B[a]P and BPA increases cancerous properties of the MCF10AT1 cell line. Importantly exposure to the two pollutants leads to a greater deleterious impact than the compounds tested individually, and our data highlight the existence of a unique functional cross-talk between AhR and GPR30 in mediating those effects. The clinical relevance of the AhR/GPR30 interplay is validated by the observation of high mRNA expression levels of these two receptors in breast cancer patients as markers of poor prognosis. Finally, this study identified AhR and GPR30 as novel targets for strategies inhibiting the development of cancer-associated properties (AIG and MFE) in early-transformed human mammary cells following long-term and low-dose exposure to B[a]P and BPA.

## Materials and Methods

### Cell Culture

Early-transformed human mammary MCF10AT1 cells and the MCF10AT1-derived cancerous MCF10CA1a.cl1 cells (Karmanos Institute, Detroit, USA) were purchased from Karmanos Institute (Detroit, USA) and maintained in complete DMEM/Ham's F12 medium with 5% horse serum (Thermo Fisher Scientific, Waltham, USA) and additional supplements: 100 ng/mL cholera enterotoxin, 10 mg/mL insulin, 0.5 mg/mL hydrocortisol, 20 ng/mL epidermal growth factor (Sigma, Saint Louis, USA) 100 units/mL penicillin and 100 mg/mL streptomycin. MCF-7 cells were purchased from ATCC (Teddington, UK) and grown according to the manufacturer's recommendations. HG5LN PXR cells stably expressing PXR (47), were grown in DMEM containing phenol red and 1 g/L glucose with 5% fetal calf serum (FCS) and additional supplements: 1 mg/mL G418 and 0.5 μg/mL puromycin.

### Reagents

B[a]P and BPA were purchased from Sigma (Saint Louis, USA). 2,3,7,8-Tetrachlorodibenzo-p-dioxin (TCDD), used as a control, was purchased from LGC Standard (Molsheim, France). The GPR30 agonist G1, the GPR30 antagonist G15, and the AhR agonist ITE were from TOCRIS Bioscience (Bristol, UK); the AhR antagonist GNF351 from Calbiochem (Billerica, USA).

### Establishment and Maintenance of the Chronically Exposed Cellular Model

MCF10AT1 cells were chronically exposed or not to 10^−10^ M of B[a]P or to 10^−10^ M BPA, alone or in combination, during 60 days (≈20 passages) in phenol red-free DMEM/Ham's F12 medium with 5% steroid-depleted, dextran-coated and charcoal-treated horse serum, containing the above-mentioned supplements (further referred to as DCC medium). Unexposed MCF10CA1a.cl1 cells were grown concomitantly in the same medium for 60 days and named MCF10CA1a.cl1_60d_. Media and treatments were changed every 2 days. Cells were frozen every 2 weeks.

### Anchorage-Independent Growth (AIG)

Anchorage-independent growth was assessed by soft agar assay as previously described (48). Single-cell suspensions (75 × 10^3^) were seeded onto soft agar, and colonies were counted after 3 weeks of incubation.

### Mammosphere Formation Efficiency (MFE)

Single-cell suspensions were seeded using non-adherent mammosphere culture conditions (49). After 7 days, primary mammospheres (first generation) were counted, collected, trypsinized, and replated for 10 days in non-adherent culture conditions to generate second-generation mammospheres. The culture media were replenished every 2–3 days.

### RNA Extraction and Real-Time Quantitative Polymerase Chain Reaction (RT-qPCR)

Total RNA extraction, reverse transcription and RT-qPCR measurements were performed as described previously (29, 48). RNA lysates were extracted after exposure to the different molecules tested, untreated cells were used as controls in the presence of the corresponding volume of solvent. One microgram of total RNA from each sample was reverse-transcribed as previously described (48). RT-qPCR measurements were performed using a CFX96 with the SsoAdvanced Universal SYBR green supermix (BioRad, Hercules, USA), according to the manufacturer's recommendations. The primers used to explore the expression of the *ER*α*, ER*β*, PXR, AhR, GPR30, CYP1A1*, and *28S* genes are listed in the Supplementary Table 1.

### Western Blot

Western blot experiments were performed as previously described (48) using the following antibodies: AhR (1:1,000, ab2770; Abcam, Paris, France), GPR30 (1:1,000, NBP1-31239; Novus Biologicals, Littleton, USA), α-tubulin (1:10,000, T5168; Sigma), phospho-p42/44 MAPK (1:1,000, 9106; Cell Signaling), p42/44 MAPK (1:1,000, 9102; Cell Signaling, Danvers, USA).

### *GPR30* or *AhR Silencing*

Stealth^TM^ siRNAs siRNA-GPR30 and Stealth^TM^ siRNAs siRNA-AhR and their corresponding scrambled control RNA (scrambled) were obtained from Ambion (Carlsbad, USA,4390825) and Invitrogen (Carlsbad, USA, AHRHSS100337/336), respectively. Fifty nM of siRNA-GPR30, 5 nM of siRNA-AhR or corresponding scrambled RNA were transfected into MCF10AT1 cells with lipofectamine RNAimax (Invitrogen). Transfections were performed directly at the time of cell seeding. Western blots were performed 48 h post-transfection. Exposure to G1, TCDD, or ITE for RNA collection or luciferase assay was performed 24 h post-transfection.

### Luciferase Assay

Cells were plated and then transfected with 150 ng XRE-firefly luciferase reporter plasmid (XRE-luc) (50) and 10 ng Renilla luciferase plasmid (pTK-RL). Twenty-four hours after transfection cells were grown for 8 h in the presence of the indicated treatment, and luciferase activity was then assessed as previously described (48).

### Short-Term Exposure Experiments

In AIG experiments, MCF10AT1 cells were exposed to BPA and/or B[a]P 10^−10^ M, ITE 10^−10^ M, or G1 10^−10^ M for 72 h in the presence or the absence of a 2 h pre-treatment with the AhR antagonist GNF351 10^−7^ M or the GPR30 antagonist G15 10^−8^ M. Exposure was maintained throughout the course of the experiments. In MFE assays, MCF10AT1 cells were exposed to BPA and/or B[a]P 10^−10^ M, ITE 10^−10^ M, or G1 10^−10^ M in the presence or the absence of GNF351 10^−7^ M or G15 10^−8^ M only during the time-course of the experiments.

### RNA-Seq Experiments and Analyses

RNA isolation, library preparation, and RNA-Seq were performed by the core facility ProfileXpert (Lyon, France) from three independent cell-culture replicates of each tested cell line (unexposed MCF10AT1_60d_ cells, B[a]P 10^−10^ M exposed MCF10AT1_60d_, BPA 10^−10^ M exposed MCF10AT1_60d_ cells, B[a]P+BPA 10^−10^ M exposed MCF10AT1_60d_). The resulting RNA were isolated using the RNeasy mini kit (Qiagen) according to the manufacturer's protocol and ribosomal depletion was performed with the Ribo-zero gold kit (Epicentre). Libraries were performed from 20 ng ribosomal depleted RNA with the NEXTFLEX® Rapid Directional RNA-Seq Library Prep Kit (BIOO-Scientific). Libraries were sequenced using an Illumina NextSeq 500 platform (flow cell highoutput V2) and a 75 bp paired-end sequencing with ~30–35 million reads per sample. After trimming, reads were aligned to the human genome (hg19) using TopHat-2 v. 2.1.0 and data normalization (FPKM) was performed with Cufflinks software v.2.1.1. Data were logged on the NCBI Gene Expression Omnibus (GEO) website (http://www.ncbi.nlm.nih.gov/geo/) and are available as a GSE142073 dataset. Transcripts were considered as differentially expressed when the *p*-value of a student non-parametric *t*-test was ≤ 0.05. The Aryl Hydrocarbon Receptor Signaling Canonical Pathway was evaluated with a functional analysis created with Ingenuity Pathway Analysis software (IPA®, QIAGEN Redwood City, www.qiagen.com/ingenuity). The GPR30 gene expression signature described by Pandey and collaborators (51) was introduced in the Ingenuity Pathway Analysis software to assess the GPR30 signaling pathway.

### Cell Proliferation

A total of 4 × 10^4^ cells/well were seeded onto and cultured in 24-well plates. Proliferating cells were analyzed using the Scepter™ 2.0 Cell Counter (Merck Millipore, Billerica, USA).

### Cell Viability Assay

A total of 10^4^ cells/well were plated onto a 96-well plate and treated for 4 days as indicated. Cell viability was assessed as previously described (52).

### Breast Tumor Cohorts

Women with primary breast tumors (*n* = 113) and known clinical follow-up who had not received any therapy before surgery and who relapsed, or not, while receiving endocrine therapy and/or chemotherapy were recruited from the BB-0033-00050, Biological Resources Center (CRB) Centre Léon Bérard, Lyon France (CLB cohort, Supplementary Table 2) (53). This study has been approved by the local ethics committee (CRB Centre Léon Bérard, France). The CRB Centre Léon Bérard is quality certified according NFS96-900 French standard and, ISO 9001 for clinical trials, ensuring scientific rigor for sample conservation, traceability and quality, as well as ethical rules observance and defined rules for transferring samples for research purposes (Ministry of Health for activities authorization n° AC-2019-3426 and DC-2008-99). The material used in the study has been collected in agreement with all applicable laws, rules, and requests of French and European government authorities, including the patients' informed written consents. Extraction of total RNA from frozen tumor samples and RT-qPCR measurements were performed as previously described (52, 53). Univariate analyses were performed using the SPSS™ Software (IBM, USA). The IBM SPSS software (IBM) was used for all statistical analyses in which the prognostic value of *AhR* and *GPR30* mRNA levels was analyzed. The data were divided at the median value of *AhR* or *GPR30* mRNA expression into two groups with either high or low expression levels. The Kaplan-Meier plotter (KMP) cohort was established from a meta-analysis of the gene-expression profiles of 1,877 primary breast cancer samples from patients who had not received any therapy prior to surgery (54). A *p* < 0.05 was considered statistically significant.

### Long-Term Inhibitory Strategies

MCF10AT1 cells were chronically (60 days) exposed or not to (B[a]P + BPA) 10^−10^ M, alone or in combination with GNF351 10^−7^ M and/or G15 10^−8^ M. Control experiments were performed in MCF10AT1 cells exposed for 60 days to GNF351 10^−7^ M and/or G15 10^−8^ M. The resulting established cells were then tested for AIG and MFE as described above.

## Results

### Chronic and Low-Dose Exposure to B[a]P and/or BPA of Early-Transformed MCF10AT1 Cells Leads to an Enhanced and Acquired Aggressive Phenotype

The MCF10AT1 cells and the MCF10AT1-derived MCF10CA1a.cl1 respectively represent the early-transformed and cancerous stages in the unique MCF10 model of triple negative breast cancer progression (43, 44). Validation of progression to malignancy of MCF10AT1 cells was investigated by assessing: (i) anchorage-independent growth (AIG), a hallmark of carcinogenesis associated with aggressiveness and metastasis in malignant cells; (ii) cancer stem-like and self-renewing properties by assessing first and second generation mammosphere-forming efficiency (MFE), as a growing body of evidence suggests that cancer stem-like cells are involved in generating and maintaining pre-malignant and malignant lesions (55–57). Consistent with the substage of breast cancer progression displayed by each cell line, MCF10AT1 cells formed significantly fewer and smaller colonies in soft agar (AIG) and significantly fewer mammospheres than the cancerous MCF10CA1a.cl1 cells (Supplementary Figures 1A,B).

**Figure 1:**
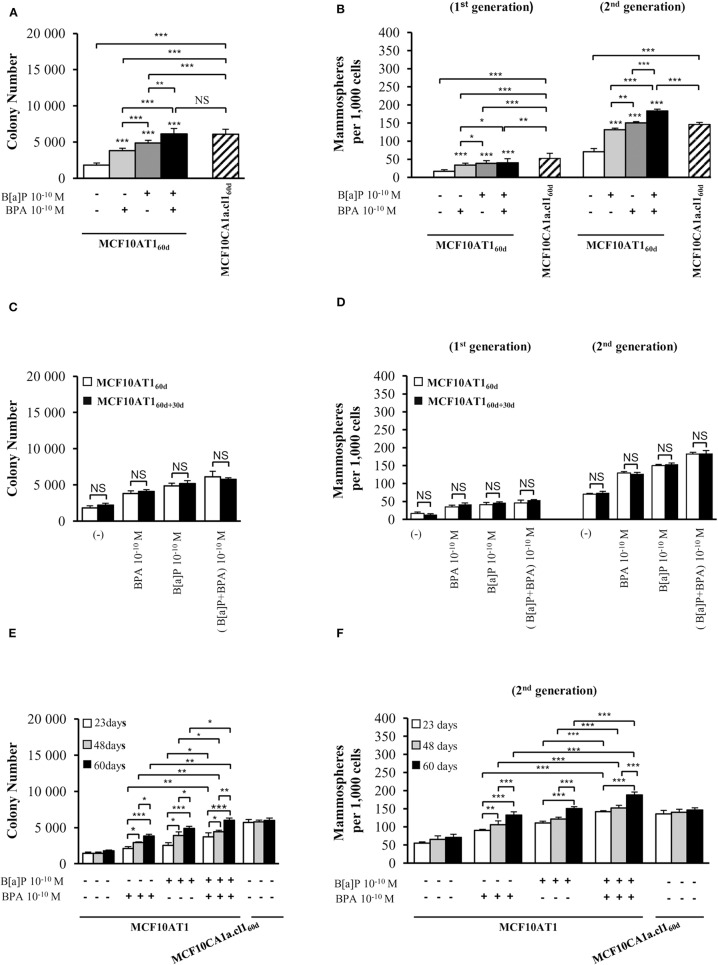
Cumulative and low-dose exposure of BPA and/or B[a]P increases MCF10AT1 cancerous properties (AIG and MFE assays). **(A)** Average number of MCF10AT1_60d_ colonies in soft agar after 60 days of chronic exposure to B[a]P and/or BPA (10^−10^ M). **(B)** Average number of primary and secondary MCF10AT1_60d_ mammospheres formed after 60 days of chronic exposure to B[a]P and/or BPA (10^−10^ M). In **(A,B)**, exposure was maintained throughout the course of the experiments. **(C,D)** After 60 days of chronic exposure to B[a]P and/or BPA (10^−10^ M), MCF10AT1 cells were grown for a further 30 days without any exposure, and single-cell suspensions of cells were seeded in soft agar **(C)** or in non-adherent mammosphere culturing conditions **(D)**. **(E,F)** Time-dependent acquisition of the aggressive phenotype: average number of colonies in soft agar **(E)** and of secondary mammospheres **(F)** following 23, 48, or 60 days of chronic exposure to B[a]P and/or BPA (10^−10^ M). Data illustrated in **(A–F)** represent mean ± SD of at least three independent experiments, in triplicate. ***p < 0.001, **p < 0.01, *p < 0.05 or NS (not significant) in Student *t*-test.

In order to identify the impact of B[a]P and BPA on progression to malignancy in conditions mimicking environmental exposure, the MCF10AT1 cells were chronically (60 days) exposed or not to physiologically-relevant concentrations (10^−10^ M) of B[a]P and/or BPA (58–62) (MCF10AT1_60d_). Unexposed MCF10AT1_60d_ and MCF10CA1a.cl1_60d_ cells displayed unmodified AIG and MFE phenotypes (Figures 1A,B and Supplementary Figure 1C) compared to the corresponding parental cells (Supplementary Figures 1A,B). MCF10AT1_60d_ cells exposed to the carcinogenic B[a]P pollutant at a concentration as low as 10^−10^ M exhibited significantly increased AIG (Figure 1A), increased colony size (Supplementary Figure 1C) and higher MFE (Figure 1B) compared to the unexposed MCF10AT1_60d_ cells. The EDC BPA (10^−10^ M, 60 days exposure) also enhanced the cancerous properties of the exposed MCF10AT1_60d_ cells (significant increase in AIG, in colony size and in MFE), while that impact was always significantly lower than that of 60-days exposure to 10^−10^ M B[a]P (Figures 1A,B, Supplementary Figure 1C). Of utmost interest, the combination of B[a]P with BPA (10^−10^ M) had a significantly greater effect than B[a]P (10^−10^ M) tested individually (Figures 1A,B, Supplementary Figure 1C). The cancerous features displayed by the MCF10AT1_60d_ cells exposed to the B[a]P + BPA (10^−10^ M) combination was at least equivalent (Figure 1A and Supplementary Figure 1C) if not greater (second generation of mammospheres, Figure 1B) than those displayed by the unexposed cancerous MCF10CA1a.cl1_60d_ cells. Supplementary Figure 2 eliminated the possibility that the aggressive phenotypes observed in the different exposed MCF10AT1_60d_ cells were the consequence of an increase in cell proliferation (compared to unexposed MF10AT1_60d_).

Unexposed or exposed MCF10AT1_60d_ cells were grown for a further 30 days in the absence of any treatment (MCF10AT1_60+30d_ cells), and then tested for AIG and MFE. The number and size of colonies in soft agar (Figure 1C and Supplementary Figure 1D) and the number of mammospheres (Figure 1D) were similar between MCF10AT1_60d_ and MCF10AT1_60+30d_ cells, thus demonstrating that the aggressive phenotype induced by chronic exposure of MCF10AT1 cells to low-doses of B[a]P and/or BPA is an acquired phenotype.

Taking advantage of the systematic freezing of exposed cells over the experimental time-course, AIG and MFE were retrospectively tested after 23 and 48 days of exposure and compared to 60 days of exposure (Figures 1E,F). The number of colonies in soft agar and mammospheres significantly increased between 23–48 and 48–60 days of chronic exposure, thus demonstrating that the longer MCF10AT1 cells are exposed to low doses of B[a]P and/or BPA, the more aggressive is their resulting phenotype. Once again, the combined exposure to B[a]P + BPA (10^−10^ M) caused more deleterious effects than those of B[a]P or BPA individually, irrespective of the exposure time.

Collectively, our results suggest that: (i) chronic, low-dose exposure to B[a]P or BPA enhances the cancerous properties of MCF10AT1 cells, with exposure to B[a]P being the most effective; (ii) the resulting aggressive phenotype was acquired and not reversed or softened when exposure was stopped; (iii) the duration of the exposure to pollutants impacted the magnitude of the aggressiveness developed by the exposed cells; (iv) combining BPA to B[a]P had more impact than exposure to B[a]P alone and was at least equivalent or higher than that of cancerous MCF10Ca1a.cl1 cells, supporting progression toward the cancerous substage.

### AhR and GPR30 are Both Expressed and Functional in MCF10AT1 Cells

**Figure 2:**
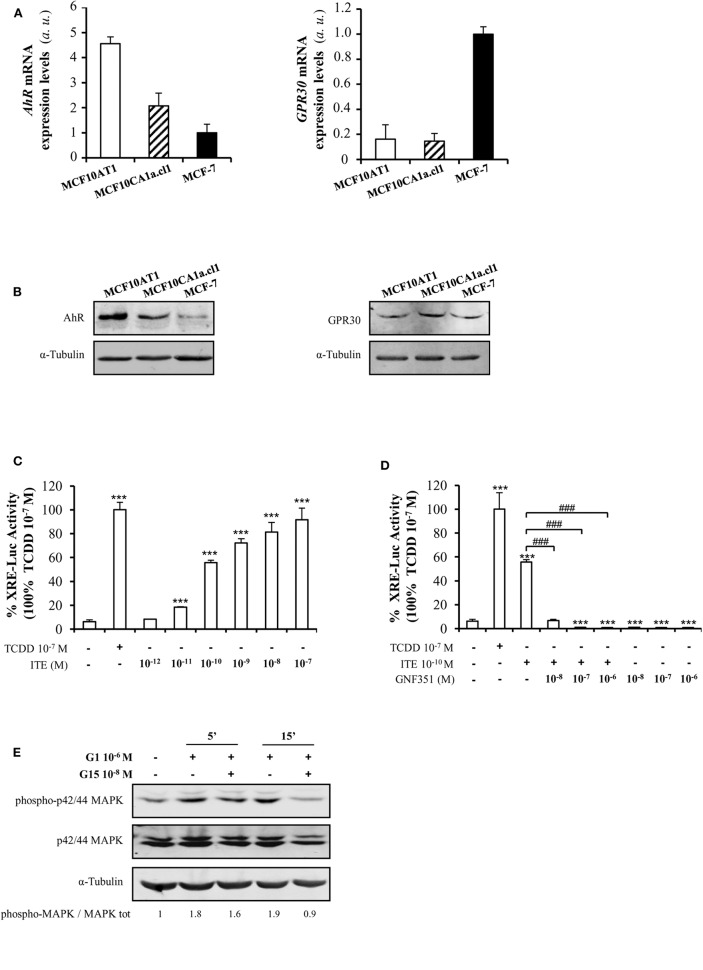
AhR and GPR30 receptors are present and functional in the MCF10AT1 cells. **(A)** RT-qPCR analysis of *AhR* and *GPR30* mRNA expression levels represented in arbitrary units *(a.u.)* in the MCF10AT1 and MCF10CA1a.cl1 cells. MCF-7 cells were used as a control. Values represent mean ± SD of three independent experiments conducted in triplicate. **(B)** Representative Western blot analyses from three independent experiments of AhR and GPR30 protein expression in MCF10AT1 and MCF10CA1a.cl1 cells. MCF-7 cells were used as a control. **(C)** XRE-luciferase activity following 8 h exposure of MCF10AT1 cells to ITE at the indicated concentrations. TCDD 10^−7^ M was used as a control and results were expressed as % of TCDD 10^−7^ M activity. ***p < 0.001 in Student *t*-test. **(D)** XRE-luciferase activity upon 8 h of exposure to ITE 10^−10^ M alone or in combination with GNF351 at the indicated concentrations. TCDD 10^−7^ M was used as a control, and results were expressed as % of TCDD 10^−7^ M activity. Student *t*-tests revealed the statistically significant differences between unexposed and exposed cells: ***p < 0.001; and between ITE and ITE+GNF351: ^###^*p* < 0.001. Values in **(C,D)** represent mean ± SD of three independent experiments. **(E)** Representative Western blot analyses from three independent experiments of the phospho-MAPK/MAPK ratio upon exposure of MCF10AT1 cells to G1 (GPR30 agonist) for the times indicated, in the presence or absence of a 2 h pre-treatment with G15 (GPR30 antagonist).

As AhR is the main target of B[a]P and BPA is known to bind to ERα, ERβ, GPR30, and PXR, we investigated the presence of these receptors in MCF10AT1 cells. Supplementary Figure 3 validated that *ER*α*, ER*β, and *PXR* were not or scarcely expressed in MCF10AT1 and MCF10CA1a.cl1 cells. Conversely, we newly reported that AhR and GPR30 receptors are concomitantly expressed at the mRNA and protein levels in MCF10AT1 and MCF10CA1.cl1 cells (Figures 2A,B). Of interest, AhR expression levels are higher both at the mRNA and protein levels in the MCF10AT1 cells compared to MCF10ACA1a.cl1.

Gene reporter experiments performed in MCF10AT1 cells demonstrated that TCDD, a well-known AhR ligand and activator of AhR-direct transcriptional activity (50), led to an increase in XRE-luciferase activity (Figure 2C). The selective AhR agonist ITE (63) also led to a stronger and dose-dependent activation of AhR-driven transcriptional activity in MCF10AT1 cells (Figure 2C). A concentration as low as 10^−11^ M of ITE was sufficient to give rise to a significant increase in XRE-luc activity (Figure 2C) and the effect of ITE was prevented (Figure 2D) by a 100-fold excess of the AhR antagonist GNF351 (64).

Rapid phosphorylation of MAPK is one of the main downstream effects of GPR30 activation (65). In MCF10AT1 cells, the MAPK pathway is rapidly activated (increase in the phospho-p42/p44 MAPK/MAPK ratio) in the presence of the selective GPR30 agonist G1 (66) and this activation is impaired in the presence of the GPR30 antagonist G15 (67) (Figure 2E), newly revealing the presence of functional GPR30 in MCF10AT1 cells.

### The MFE and AIG Responses Triggered by B[a]P and/or BPA Occur Through a Functional Cross-Talk Between AhR and GPR30 in MCF10AT1 Cells

We then aimed at investigating the possible involvement of the AhR and/or GPR30 receptors in mediating the tumorigenic effects of exposure to B[a]P and/or BPA 10^−10^ M in short-term experiments. This was tested in the presence or absence of selective antagonists of AhR or GPR30 (GNF351 and G15, respectively) at non-toxic concentrations (Supplementary Figures 4A,B).

**Figure 3:**
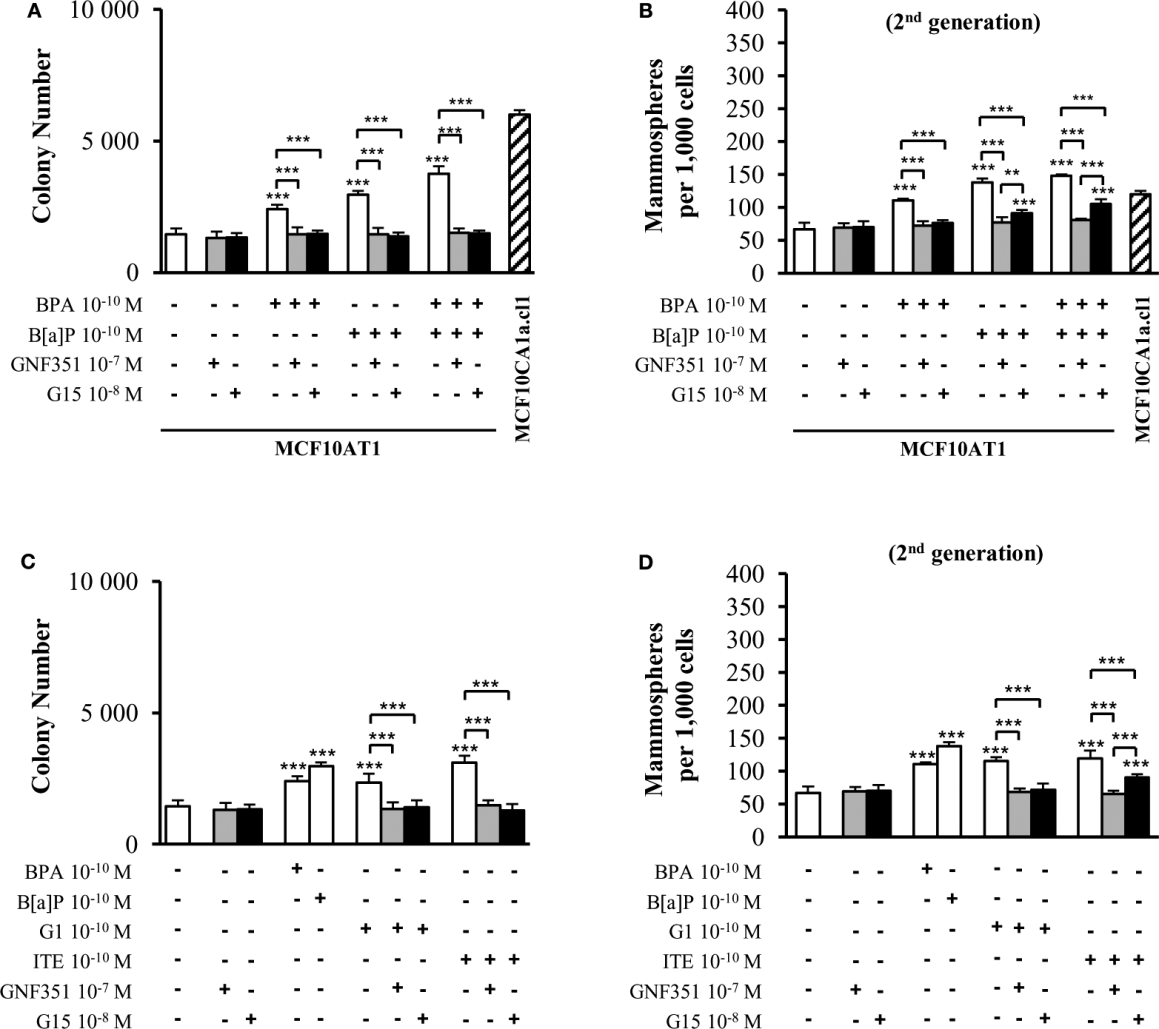
Effects of short-term exposure of MCF10AT1 cells by BPA, B[a]P, ITE, and G1 on AIG and MFE are inhibited by GPR30 and AhR antagonists. Average number of colonies in soft agar **(A)** or of secondary mammospheres **(B)** upon exposure to B[a]P and/or BPA 10^−10^ M for 72 h in the presence or absence of a 2 h pre-treatment with GNF351 10^−7^ M or G15 10^−8^ M. Exposure was maintained throughout the course of the experiments. Unexposed MCF10CA1a.cl1 cells were used as a control. **(C)** Average number of colonies in soft agar or **(D)** secondary mammospheres formed after 72 h exposure with ITE 10^−10^ M or G1 10^−10^ M in the presence or the absence of a 2 h pre-treatment with GNF351 10^−7^ M or G15 10^−8^ M. Exposure to BPA 10^−10^ M or B[a]P 10^−10^ M was used as controls. Exposure was maintained throughout the course of the experiments. Data illustrated in **(A–D)** represent mean ± SD of at least three independent experiments, in triplicate. ***p < 0.001, **p < 0.01 in Student *t*-test.

Figure 3A demonstrates that short-term (72 h) exposure to 10^−10^ M BPA and/or 10^−10^ M B[a]P were sufficient to generate increased colony numbers in AIG assays. GNF351 and G15 were able to completely inhibit the effects of B[a]P and/or BPA on AIG (Figure 3A). This result was, at least to some extent, expected with the B[a]P/GNF351 and the BPA/G15 combinations, but was totally surprising concerning the BPA/GNF351 and the B[a]P/G15 combinations. In MFE assays conducted with MCF10AT1 cells exposed only during the time-course of the experiments (Figure 3B), the increased number of mammospheres induced by 10^−10^ M BPA was totally blocked in the presence of G15, as expected, but also in the presence of GNF351. Conversely, B[a]P-induced MFE was completely inhibited by GNF351, while the GPR30 antagonist G15, surprisingly resulted in a partial but significant inhibition. The impact of the G15 or GNF351 on the combination B[a]P+BPA was similar to that observed with B[a]P alone.

We then performed the same experiments using G1 and ITE agonists, at 10^−10^ M non-toxic concentrations (Supplementary Figures 4C,D). Figures 3C,D show similar data as those obtained with B[a]P and/or BPA in Figures 3A,B, namely that: (i) low-dose exposure of MCF10AT1 cells to 10^−10^ M G1 or 10^−10^ M ITE gives rise to significantly increased colony number (AIG) or increased MFE in the same range as that observed with 10^−10^ M BPA or 10^−10^ M B[a]P, respectively; (ii) G15 and GNF351 were both able to totally inhibit G1-dependent AIG or MFE; (iii) the impact of ITE on MFE was totally blocked in the presence of GNF351, while only partially inhibited by G15.

**Figure 4:**
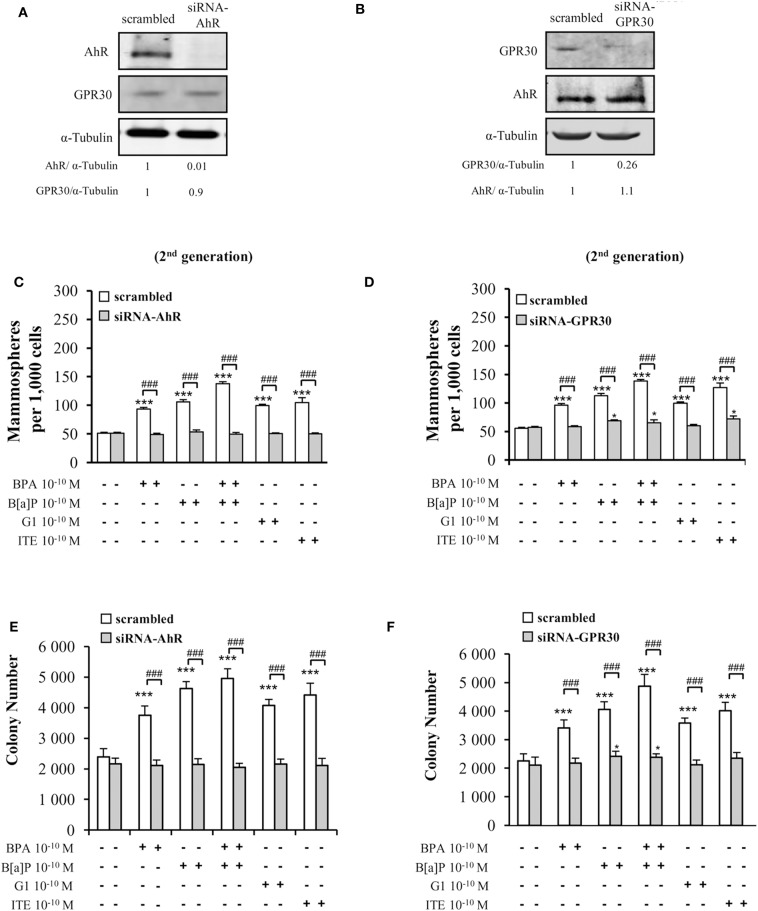
Effects of short-term exposure of BPA, B[a]P, ITE and G1 10^−10^ M on AIG and MFE are inhibited by siRNA-AhR and siRNA-GPR30. Representative Western blot analysis from three independent experiments of AhR and GPR30 expression in transfected MCF10AT1 cells with **(A)** siRNA-AhR, **(B)** siRNA-GPR30 or their scrambled controls. Quantification of protein expression levels was normalized against tubulin expression. **(C,D)** Secondary mammospheres formation and **(E,F)** average number of colonies in soft agar, with the following treatments: BPA and/or B[a]P, G1, or ITE, 10^−10^ M. Cells were transfected with either siRNA-AhR, siRNA-GPR30 or their scrambled controls before being subjected to the treatments. Treatments were maintained throughout the course of experiments. (mean ± SD of 2 independent experiments, in triplicate). ***p < 0.001, *p < 0.05 vs. their respective unexposed; ^###^*p* < 0.001 siRNA vs. scrambled in Student *t*-test.

To confirm the above data supporting the involvement of both GPR30 and AhR receptors in mediating the effects of low dose (10^−10^ M) BPA, B[a]P, G1, or ITE, we used a siRNA strategy silencing either AhR or GPR30 (Figures 4A,B). We verified that siRNA-AhR or siRNA-GPR30 had, however, no impact on GPR30 or AhR expression, respectively (Figures 4A,B), nor on MCF10AT1 cell viability (Supplementary Figures 4E,F).

Findings from AIG and MFE experiments conducted in MCF10AT1 exposed to BPA, B[a]P, G1, or ITE 10^−10^ M in the presence of siRNA-AhR or of siRNA-GPR30 (Figures 4C–F) corroborated those obtained using the AhR- or GPR30-antagonists (GNF351 and G15, respectively) (Figures 3A–D). Indeed, AhR knock-out resulted in a total inhibition of BPA-, B[a]P-, G1-, or ITE-mediated effects (Figures 4C,E). GPR30 silencing, while totally inhibiting BPA- or G1-mediated effects (Figures 4D,F), was slightly less effective in inhibiting MFE or AIG assays when cells were exposed to B[a]P (Figures 4D,F) or ITE (Figure 4D). The impact of siRNA-AhR or siRNA-GPR30 on the combination B[a]P+BPA was similar to that observed with B[a]P alone (Figures 4C–F).

Altogether these results support the idea that both AhR and GPR30 receptors play a role in mediating the deleterious effects exerted by BPA, G1, B[a]P, or ITE on the MCF10AT1 early-transformed cells. More importantly, our data highlight a functional cross-talk between GPR30 and AhR.

### GPR30-Dependent Mechanisms are Correlated With Enhanced AhR Transcriptional Activity

**Figure 5:**
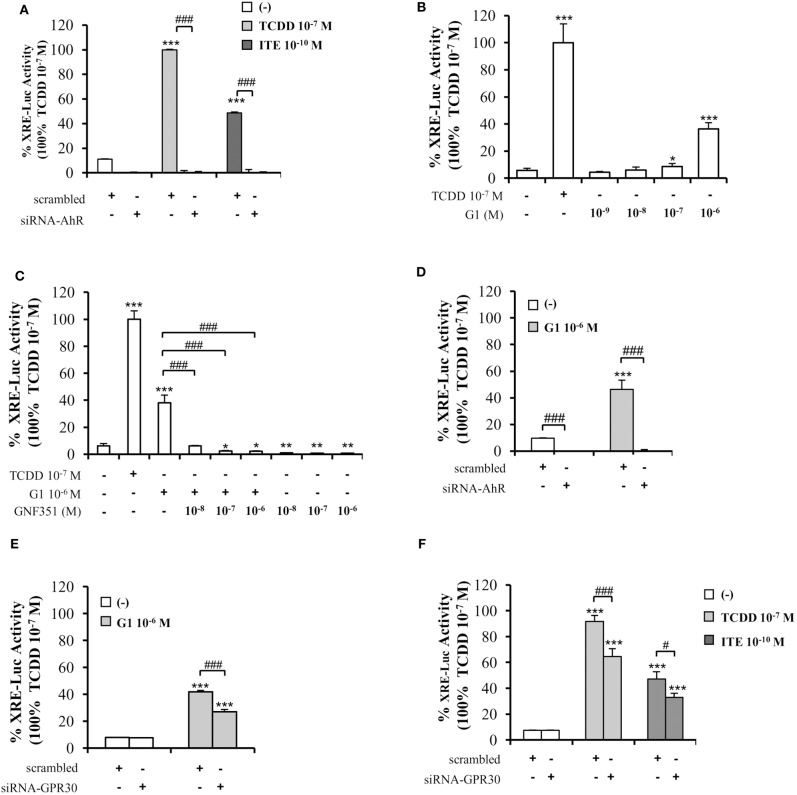
Involvement of GPR30 in AhR transcriptional activity using XRE-luciferase assays. XRE-luciferase activity following 8 h exposure of MCF10AT1 cells to **(A)** ITE at the indicated concentrations, TCDD 10^−7^ M was used as a control and results were expressed as % of TCDD 10^−7^ M activity; in the presence or absence of siRNA-AhR **(B)** G1 at the indicated concentrations. TCDD 10^−7^ M was used as a control; **(C)** G1 10^−6^ M alone or in combination with GNF351 at the indicated concentrations; TCDD 10^−7^ M was used as a control. **(D,E)** XRE-luciferase activity in MCF10AT1 cells transfected with **(D)** siRNA-AhR, **(E)** siRNA-GPR30 or their respective scrambled and treated 48 h after siRNA transfection for 8 h with G1 10^−6^ M. **(F)** XRE-luciferase activity in MCF10AT1 cells transfected with either scrambled RNA or siRNA-GPR30 and treated for 8 h with TCDD 10^−7^ M or ITE 10^−10^ M after 48 h of siRNA transfection. All data represent mean ± SD of three independent experiments conducted in triplicate. *p < 0.05, **p < 0.01, ***p < 0.001; ^#^*p* < 0.05; ^###^*p* < 0.001 in Student *t*-test.

To further decipher the interplay between AhR and GPR30, we performed XRE-luciferase reporter assays. As anticipated, AhR-driven transcriptional activity in MCF10AT1 cells was fostered by TCDD 10^−7^ M or ITE 10^−10^ M and lost following AhR silencing (Figure 5A). Very interestingly, the GPR30 agonist G1 was also able to significantly increase XRE-luciferase activity in a dose-dependent manner, as illustrated by its effect at 10^−7^ M and 10^−6^ M, which is, to our knowledge, the first such report in the literature (Figure 5B). The G1-induced XRE-luciferase signal was totally inhibited in the presence of GNF351 (Figure 5C) and also in the presence of siRNA-AhR (Figure 5D). Silencing GPR30 by a siRNA-GPR30 strategy gave rise to a partial but significant decrease in G1- (10^−6^ M) (Figure 5E), TCDD- (10^−7^ M), and ITE- (10^−10^ M) (Figure 5F) -dependent activation of XRE-luciferase activity. Altogether, these data recapitulate the cross-talk between AhR and GPR30 on a simplified XRE-luciferase response.

**Figure 6:**
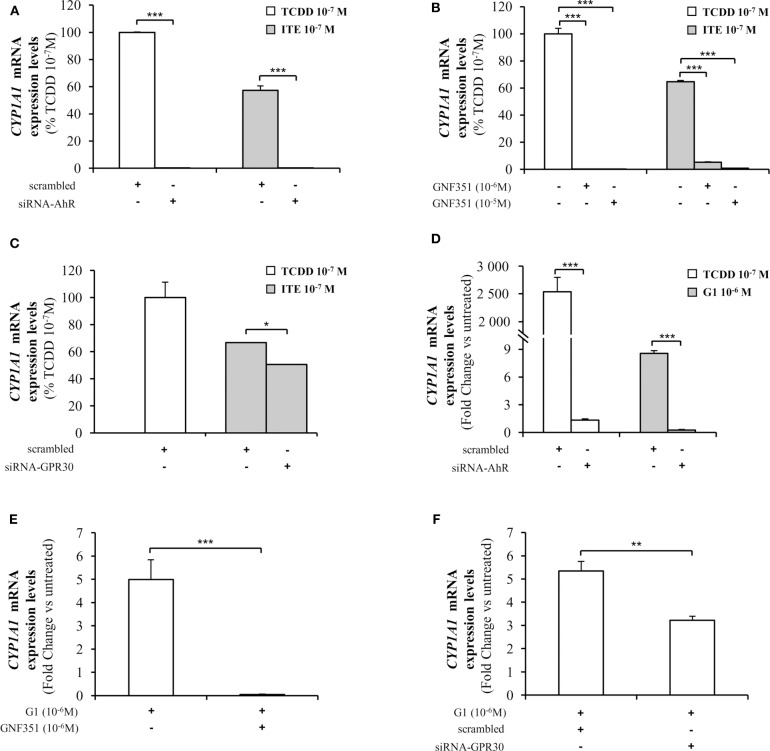
Analysis of *CYP1A1* mRNA levels by quantitative RT-qPCR in MCF10AT1 cells. **(A)** *CYP1A1* expression levels in cells transfected with either siRNA-AhR or scrambled RNA after 4 h of exposure to ITE 10^−7^ M. TCDD 10^−7^ M was used as a control and results are expressed as % of TCDD 10^−7^ M activity. **(B)** *CYP1A1* mRNA expression levels in cells treated with GNF351 alone at indicated concentrations or in combination with ITE 10^−7^ M or TCDD 10^−7^ M. TCDD 10^−7^ M was used as a control and results are expressed as % of TCDD 10^−7^ M activity. **(C)** *CYP1A1* mRNA expression levels in cells transfected with either siRNA-GPR30 or scrambled RNA and exposed to ITE 10^−7^ M for 4 h. TCDD 10^−7^ M was used as a control and results are expressed as % of TCDD 10^−7^ M activity. **(D)** *CYP1A1* mRNA expression levels in cells transfected with either siRNA-AhR or scrambled RNA and exposed to G1 10^−6^ M or TCDD 10^−7^ M for 24 h. TCDD 10^−7^ M was used as a control. **(E)** *CYP1A1* mRNA expression levels in cells treated with G1 10^−6^ M in the presence or absence of GNF351 10^−6^ M. **(F)** *CYP1A1* mRNA expression levels in cells transfected with either siRNA-GPR30 or scrambled RNA and exposed to G1 10^−6^ M for 24 h. Data represent mean ± SD of 3 independent experiments conducted in triplicate. **(A–F)** The Student *t*-test was applied to reveal statistically significant differences between treatments: *p < 0.05, **p < 0.01, ***p < 0.001.

As *CYP1A1* is amongst the canonical genes regulated by AhR with a promoter containing typical XRE, we further investigated endogenous *CYP1A1* mRNA levels in MCF10AT1 cells upon ITE and G1 treatment. Consistent with Figures 5, 6 revealed that: (i) *CYP1A1* mRNA levels significantly increased upon treatment with TCDD 10^−7^ M and ITE 10^−7^ M (Figure 6A); (ii) the impact of ITE 10^−7^ M was completely impaired in the presence of siRNA-AhR (Figure 6A) or GNF351 10^−6^ M (Figure 6B), and significantly decreased in the presence of siRNA-GPR30 (Figure 6C). Figures 6D–F highlight that G1 10^−6^ M resulted in a significant increase in *CYP1A1* mRNA levels, and that this was totally impeded in the presence of siRNA-AhR (Figure 6D), GNF351 10^−6^ M (Figure 6E) and significantly decreased in the presence of siRNA-GPR30 (Figure 6F). Altogether, our XRE-luciferase experiments and RT-qPCR experiments confirmed, at the transcriptional level, that GPR30-dependent mechanisms significantly impact and favor, directly or indirectly, AhR-driven transcriptional-dependent events.

Having verified that protein expression levels of AhR and of GPR30 were similar in both the unexposed and the pollutants-exposed MCF10AT1_60d_ cells (Supplementary Figure 5A), the RNAseq data of each cell line were analyzed using the Ingenuity Pathway Analysis software. AhR core signaling was identified as significantly dysregulated in the MCF10AT1_60d_ exposed cells compared to unexposed control MCF10AT1_60d_ cells. Indeed, the significant enrichment of this canonical pathway was observed both in the B[a]P-exposed MCF10AT1_60d_ cells (*p* = 9.39 10^−3^), in the B[a]P + BPA exposed MCF10AT1_60d_ cells (*p* = 7.56 10^−3^), but also in the BPA-exposed MCF10AT1_60d_ cells (*p* = 1.26 10^−2^) (data not shown). Conversely, assessing the GPR30 signature previously described by Pandey and collaborators (51) in the Ingenuity Pathway Analysis software did not reveal any dysregulation in GPR30 signaling when the exposed MCF10AT1_60d_ cells were compared with the unexposed control MCF10AT1_60d_ cells (data not shown). As rapid phosphorylation of MAPK is one of the main downstream effects of GPR30 activation (65), we assessed the activation status of the MAPK pathway in the unexposed and exposed MCF10AT1_60d_ cells. Supplementary Figure 5B demonstrated that chronic and low-dose-exposure of MCF10AT1 cells to B[a]P and/or BPA at 10^−10^ M did not lead to any activation of p42/p44MAPK (MCF1Ca1.cl1_60d_ cells were used as controls). Altogether, our data suggest that AhR signaling is constitutively activated in the MCF10AT1_60d_ exposed to pollutants vs. unexposed cells.

### The *GPR30/AhR* Gene Expression Signature Indicates Poor Prognosis

**Figure 7:**
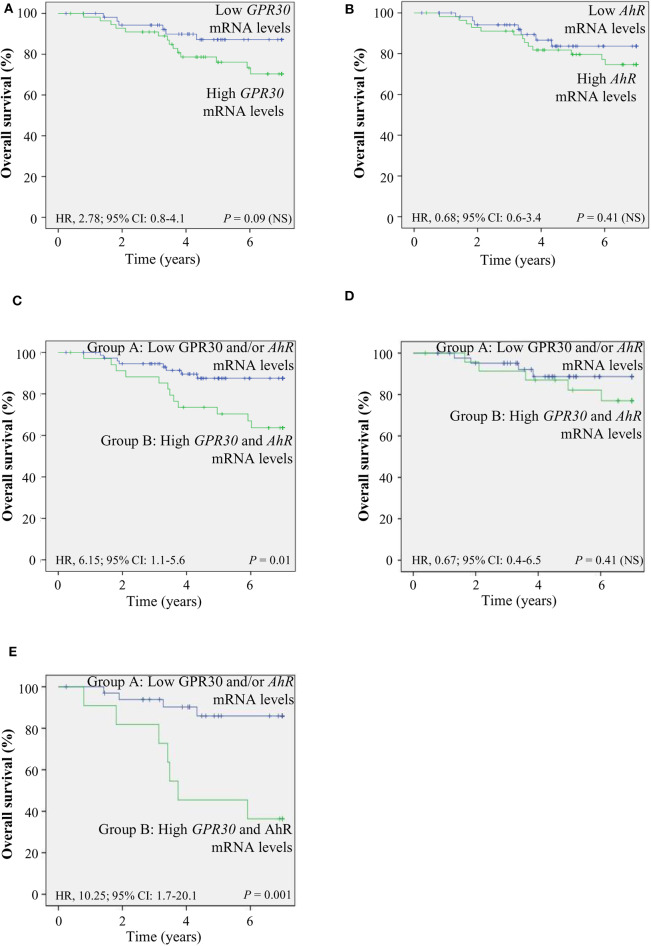
The *AhR/GPR30* mRNA expression signature is of poor prognosis and is associated with shorter overall survival (OS). Kaplan-Meier analysis (univariate analysis) of OS in the CLB breast cancer cohort of: **(A)** *GPR30* mRNA expression levels; **(B)** *AhR* mRNA expression levels; **(C–E)** Patients were divided into two groups: patients expressing low *GPR30* mRNA levels and/or low levels of *AhR* mRNA (group A) and patients expressing high mRNA levels of *GPR30* and *AhR* (group B). **(C)** *AhR/GPR30* gene expression signature in all breast tumor samples. **(D)** *GPR30/AhR* gene expression signature in the ER-positive subclass. **(E)** *GPR30/AhR* mRNA expression signature in the ER-negative subclass. NS, not significant.

To investigate the clinical relevance of AhR and GPR30, we performed RT-qPCR analyses to explore *GPR30* and *AhR* mRNA expression levels in a cohort of 113 human primary breast tumor samples (Table 1) (53). The resulting Kaplan-Meier curves are shown in Figure 7. By univariate analysis, we found that neither *GPR30* nor *AhR* mRNA levels were informative (*p* = 0.09 and *p* = 0.41, Figures 7A,B, respectively), while an *AhR/GPR30* gene expression signature based on high expression levels of both *GPR30* and *AhR* was significantly associated with shorter overall survival (OS) (*p* = 0.01, Table 1 and Figure 7C). Regarding breast cancer subclasses, the *AhR/GPR30* gene expression signature was more informative in the ER-negative (*p* = 0.001) than in the ER-positive (*p* = 0.41) (Table 1, Figures 7D,E) or luminal subclasses and (*p* = 0.50) (Table 1). The situation was less clear for the HER2-enriched and triple-negative subclasses, considering the limited size of available samples (*n* =16 and *n* = 21, respectively). We thus performed retrospective analysis of gene-expression array data using the KMP cohort, which contains a sizeable number of breast cancer patients (1,308 ER-positive, 569 ER-negative, 1,055 luminal, 419 HER2-enriched, and 403 triple-negative/basal-like). The most striking results (Table 2, univariate analysis) indicated that the *AhR/GPR30* signature was again more informative than *GPR30* mRNA or *AhR* mRNA levels alone and was associated with shorter OS in the ER-negative subclass (*p* = 0.005), but not in the ER-positive or the luminal subclasses (Table 2). The “all breast tumor samples” univariate analysis of the KMP cohort did not validate what was observed in the CLB cohort, but this discrepancy might reflect the difference in proportion of the ER-positive and ER-negative subclasses in the two cohorts. Finally, the KMP cohort revealed that the *AhR/GPR30* signature was associated with shorter OS in the triple-negative subclass (*p* = 0.033). Altogether, our data reveal a new/original signature based on a combination of high *AhR* mRNA expression levels and high *GPR30* mRNA expression levels that represents a novel marker for poor prognosis in breast cancer, especially in ER-negative or triple-negative subclasses.

**Table 1:** Univariate analysis of the *GPR30* mRNA expression levels, the *AhR* mRNA expression levels and the *GPR30/AhR* mRNA expression signature with regards to overall survival (OS) in different subclasses of the 113 breast cancer samples of the CLB cohort.

	***n***	***GPR30*** **mRNA levels**			***AhR*** **mRNA levels**			***AhR/GPR30*** **signature**		
		**HR^**a**^**	**95% CI^**b**^**	***p*^**c**^**	**HR^**a**^**	**95% CI^**b**^**	***p*^**c**^**	**HR^**a**^**	**95% CI^**b**^**	***p*^**c**^**
All breast tumor samples	113	2.78	0.8 to 4.1	NS (0.09)	0.68	0.6 to 3.4	NS (0.41)	6.15	1.1 to 5.6	**0.01**
ER+ subclass	68	0.66	0.4 to 9.2	NS (0.42)	0.10	0.2 to 3.1	NS (0.75)	0.67	0.4 to 6.5	NS (0.41)
ER- subclass	45	5.39	1.1 to 13.3	**0.02**	2.08	0.7 to 9.7	NS (0.15)	10.25	1.7 to 20.1	**0.001**
Luminal subclass (ER+/PR+)^d^	62	0.03	0.2 to 6.3	NS (0.87)	0.11	0.2 to 3.8	NS (0.74)	0.45	0.4 to 8.5	NS (0.50)
HER2- enriched subclass (ER-/PR-/HER2+)^d^	16	2.56	0.5 to 63.1	NS (0.11)	0.17	0.2 to 18.2	NS (0.68)	4.47	0.8 to 96.6	**0.03**
Triple negative subclass (ER-/PR-/HER2-)^d^	21	-	NA^e^	^−^	0.005	0.1 to 12.1	NS (0.94)	-	N/A^f^	-

a *HR, Hazard ratio*.

b *95% CI, 95% confidence interval*.

c *p was considered significant when p < 0.05 (bold values). NS, not significant*.

d *subclasses of breast cancer were determined using immunohistology (ER, PR, HER2) according to the St Gallen recommendation (68)*.

e *N/A, not applicable as all the cases are censored in the high GPR30 mRNA level group*.

f *N/A, not applicable as all the cases are censored in the high GPR30 and/or AhR mRNA level group*.

**Table 2:** Univariate analysis of the *GPR30* mRNA expression levels, the *AhR* mRNA expression levels and the *GPR30/AhR* mRNA expression signature with regards to overall survival (OS) in different subclasses of the 1,877 breast cancer samples of the Kaplan-Meier plotter (KMP) cohort.

	***n***	***GPR30*** **mRNA levels**			***AhR*** **mRNA levels**			***AhR/GPR30*** **signature**		
		**HR^**a**^**	**95% CI^**b**^**	***p*^**c**^**	**HR^**a**^**	**95% CI^**b**^**	***p*^**c**^**	**HR^**a**^**	**95% CI^**b**^**	***p*^**c**^**
All breast tumor samples	1,877	0.83	0.68 to 1.01	NS (0.06)	0.91	0.73 to 1.15	NS (0.4)	0.78	0.46 to 1.10	NS (0.14)
ER+ subclass	1,308	0.82	0.62 to 1.08	NS (0.15)	0.83	0.63 to 1.1	NS (0.2)	0.67	0.26 to 1.08	NS (0.06)
ER- subclass	569	1.46	1.06 to 2.02	**0.02**	1.24	0.89 to 1.73	NS (0.2)	1.88	1.43 to 2.33	**0.005**
Luminal subclass	1,055	1.2	0.88 to 1.64	NS (0.24)	0.8	0.58 to 1.11	NS (0.18)	0.95	0.5 to 1.4	NS (0.82)
HER2- enriched subclass	419	1.22	0.84 to 1.77	NS (0.29)	0.79	0.54 to 1.16	NS (0.23)	0.99	0.45 to 1.53	NS (0.99)
Triple negative / Basal-like subclass	403	1.44	0.97 to 2.13	NS (0.06)	1.22	0.79 to 1.88	NS (0.36)	1.85	1.28 to 2.41	**0.033**

a *HR, Hazard ratio*.

b *95% CI, 95% confidence interval*.

c *p was considered significant when p < 0.05 (bold values). NS, not significant*.

d *breast cancer subclasses were based on the St Gallen recommendation (68) according to Gyorffy et al. (69)*.

### Strategies Inhibiting the Impact of Chronic and Low-Dose Exposure to B[a]P and BPA in Early-Transformed MCF10AT1 Cells

**Figure 8:**
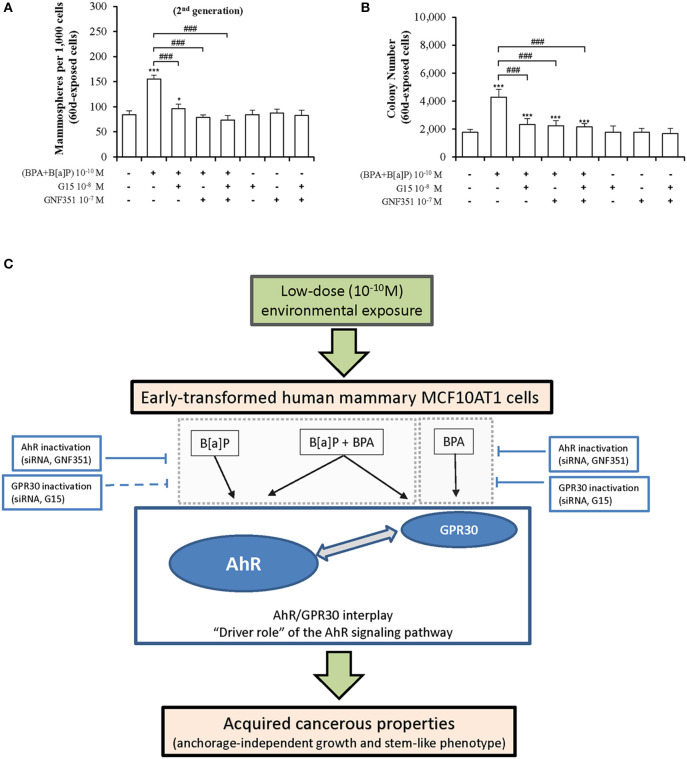
Long-term and low-dose exposure to B[a]P and/or BPA in promoting cancerous properties of early-transformed human mammary cells is driven by AhR/GPR30 cross-talk. Inhibition of the effects due to chronic and low-dose exposure of MCF10AT1 cells to BPA+B[a]P (10^−10^ M), using AhR and/or GPR30 antagonists: **(A)** formation of secondary mammospheres, **(B)** AIG. The MCF10AT1 cells were chronically (60 days) unexposed or exposed to B[a]P + BPA 10^−10^ M, alone or in combination with GNF351 10^−7^ M and/or G15 10^−8^ M. Control experiments were performed in MCF10AT1 cells exposed for 60 days to GNF351 10^−7^ M and/or G15 10^−8^ M (mean ± SD of 3 independent experiments, in triplicate). *p < 0.05, ***p < 0.001 vs. unexposed; ^###^*p* < 0.001 vs. BPA+B[a]P (10^−10^ M); **(C)** Diagram summarizing our findings.

To identify candidate strategies capable of blocking mammary carcinogenesis associated with chronic exposure to low doses (10^−10^M) of the environmental pollutants B[a]P and BPA, MCF10AT1 cells were exposed for 60 days, in the presence or the absence of the AhR antagonist GNF351 10^−7^ M and/or the GPR30 antagonist G15 10^−8^ M (Figures 8A,B). Exposure for 60 days to the two antagonists, alone or in combination, had no impact on MFE and AIG. Strikingly, co-exposure for 60 days to B[a]P + BPA 10^−10^ M with GNF351 10^−7^ M and/or G15 10^−8^ M was sufficient to inhibit the development of pollutants-driven enhancement of cancerous properties (AIG and MFE) in MCF10AT1 cells (Figures 8A,B).

## Discussion

A growing body of *in vitro* and *in vivo* experimental evidence suggests the implication of environmental factors in the development and progression of breast cancer. People are chronically exposed to a mixture of environmental factors, usually present at low-dose concentrations, constituting a complex exposome. B[a]P is detected at picomolar concentrations in body fluids and tissues of cancer patients (58–60). BPA is detected at nanomolar concentrations in human samples such as serum, urine and maternal milk (61, 62). The major obstacles in studying the impact of these two pollutants *in vitro* on breast tumorigenesis are thus the choice of a relevant cellular model and the use of relevant exposure conditions (long-term and low-dose exposure) mimicking natural exposure. The few studies having investigated the impact of chronic and low-dose exposure of B[a]P or BAP on tumor progression have focused on the early stage of carcinogenesis (non-transformed epithelial cells) (9–13, 29) MCF10AT1 breast cells (representing the early-transformed stage of the unique MCF10 model of mammary progression from normal epithelium to triple negative breast cancer (43, 44) have, to our knowledge, never been used to test the impact of chronic and low-dose exposure to environmental pollutants. While the genotoxic and pro-carcinogenic B[a]P and the EDC BPA are two of the most studied pollutants, no previous studies, to our knowledge, have investigated whether the combination of BPA and B[a]P, each compound possessing distinct mechanisms of action, induces potentiating effects on tumor progression.

The present study aimed at addressing: (i) the effects of long-term, cumulative exposure to low doses of B[a]P or BPA on the mammary early-transformed substage; (ii) whether the combination of BPA with B[a]P, each compound possessing distinct mechanisms of action, impacts breast tumor progression differently from what might be observed with each compound tested alone. Our study reveals that long term and low dose (10^−10^ M) exposure to B[a]P and/or BPA increases the cancerous properties (AIG and cancer stem-like properties) of the early-transformed MCF10AT1 cells, and the longer the cells were exposed, the greater was the impact. Co-exposure of MCF10AT1 cells with the B[a]P and BPA led to a significantly greater aggressive phenotype compared to B[a]P alone, suggesting that BPA facilitates the pro-carcinogenic activity of B[a]P and supporting the potentiating effects of distinct pollutants present in the exposome. Importantly, the aggressiveness developed by the exposed MCF10AT1_60d_ cells was acquired and not softened or reverted after stopping B[a]P and/or BPA exposure. Altogether, our data reveal that long-term and low dose exposure to B[a]P and/or BPA irreversibly favors the evolution of early-transformed human mammary cells toward breast tumor progression.

Mechanistically, while B[a]P and BPA are well-known activators of AhR and GPR30, respectively, our short-term exposure experiments highlight that the MFE and AIG response triggered by exposure of MCF10AT1 cells to B[a]P and/or BPA occurs through a functional cross-talk between AhR and GPR30. Regarding the impact of inactivating AhR or GPR30 by two different strategies (using an antagonist molecule or a siRNA strategy), the BPA, BPA+B[a]P, or G1 impact on AIG and MFE was totally reversed when AhR was inhibited, while the GPR30 inactivation globally seemed to lead to a significant but not total, reversion of B[a]P, BPA+B[a]P, or ITE impact (in particular in MFE experiments). Finally, this study provided evidence that long-term (60 days) co-exposure to B[a]P + BPA 10^−10^ M with the AhR antagonist GNF351 10^−7^ M and/or the GPR30 antagonist G15 10^−8^ M inhibited the development of pollutants-driven enhancement of cancerous properties in MCF10AT1 cells.

The AhR/CYP1A1 pathway participates in carcinogenesis by mediating stem properties, the formation of mammospheres, expansion of breast cancer stem cells, and the transcriptional activity of AhR is mainly implicated in such effects (24). Our study also reveals that GPR30 participates and favors AhR-dependent transcriptional activity in early-transformed breast cells. Indeed, the GPR30 agonist G1 was able to stimulate AhR-dependent driven activity and *CYP1A1* gene transcription. GPR30 silencing by a siRNA strategy led to a significant, yet moderate, decrease in ITE-, G1-, or TCDD-mediated activation of AhR-driven transcriptional activity or *CYP1A1* transcription. This suggests that GPR30-dependent mechanisms other than those influencing the AhR-driven transcriptional mechanisms might be involved in the AhR/GPR30 cross-talk impacting AIG and MFE. Altogether, our *in vitro* data support a model in which GPR30 is involved in an AhR-dependent network leading to increased cancerous properties of early-transformed mammary cells.

We thus propose a model (Figure 8C) in which AhR signaling plays a “driving role” in the AhR/GPR30 cross-talk in mediating the effects of long-term and low dose exposure of B[a]P and/or BPA on AIG and MFE in MCF10AT1 cells. The relevance of our model was supported by our RNAseq data demonstrating that the canonical AhR signaling pathway was significantly enriched in the B[a]P-exposed MCF10AT1_60d_ cells, in the B[a]P + BPA exposed MCF10AT160d cells, but also in the BPA-exposed MCF10AT1_60d_ cells. Strengthening our findings, previous studies support a role for AhR and breast tumor progression: (i) AhR expression levels were significantly up-regulated in human breast ductal carcinoma *in situ* and breast cancer tissues compared to normal/benign breast tissues (70, 71); (ii) an *in vivo* model of breast tumorigenesis suggests that AhR is constitutively activated at early stages of mammary tumorigenesis (72); (iii) the prognostic value of AhR seems to be dependent on the activation/inactivation of metastatic processes (73).

While this research was ongoing, a study was published supporting our data by demonstrating in the ERα-negative SKBR3 breast cancer cells that the environmental pollutant 3-methylcholantrene, mainly known to exert its carcinogenic effects through AhR, stimulates cell growth response through a functional interaction between AhR and GPR30 (74). However, in the ERα-positive MCF-7 breast cancer cell line, possessing the well-identified close cross-talk between AhR and ERα [for review (75)], 10^−5^ M G1 was demonstrated to increase transcription of *CYP1A1* mRNA in AhR-dependent, but GPR30-independent, mechanisms (76). As MCF10AT1 cells used in this study are triple-negative cells and the SKBR3 cells are ERα-negative, one cannot exclude that the detrimental functional cross-talk between AhR and GPR30 might be exacerbated in such a specific cellular breast context. Supporting previous data highlighted that in the ERα-negative/triple negative context, an AhR-signaling reinforces cell aggressiveness and induces breast cancer stem cells (77–80).

Altogether, our *in vitro* data thus demonstrated the role of the AhR/GPR30 cross-talk in favoring tumor progression, at least in a triple-negative breast context. Previous controversial studies emerged regarding the prognostic value of AhR or GPR30 expression levels in breast cancers (71, 73, 80–84), and these discrepancies have been suggested as possibly related to the breast cancer subgroup or the substage considered. In the present study, the clinical relevance of our *in vitro* findings was further reinforced by retrospective analysis of two independent breast cancer cohorts, showing that in only ER-negative or triple-negative breast cancer subclasses, the gene signature involving both *AhR* and *GPR30* mRNA levels were of poor prognosis.

Hence, we have provided novel insights into the progression of early-transformed human mammary cells upon long-term and low-dose exposure to B[a]P and/or BPA and deciphered the involvement of the functional crosstalk occurring between AhR and GPR30 leading to an exacerbated AhR-driven network. Our *in vitro* and retrospective data analyses further support the idea that the deleterious impact of this cross-talk might be of utmost importance in the progression of ER-negative or triple-negative breast cancers. More importantly, strategies targeting AhR and/or GPR30 were demonstrated to be efficient in inhibiting the deleterious impact of cumulative and low-dose exposure to B[a]P and/or BPA in early-transformed MCF10AT1 cells. The identification of such molecular mechanisms may help in the discovery of human biomarkers of environmental carcinogen exposure and the development of preventive strategies.

## Data Availability Statement

The datasets generated from this study can be found in the NCBI Gene Expression Omnibus (GEO) [http://www.ncbi.nlm.nih.gov/geo/ (GSE142073)].

## Ethics Statement

This study has been approved by the local ethics committee (CRB Centre Léon Bérard, France). All subjects gave written informed consent.

## Author Contributions

PAC, VM-S, VC, and BF participated in the design of the study. CD, ME, SG, and SA performed the *in vitro* data. PAC and CD performed the biostatistical analysis on the CLB cohort. BG performed the biostatistical analysis on the KMP cohort. AW, SC, and JL performed and analyzed the RNAseq data. SG, MD-A, AE, and PC participated in scientific discussions. PAC, CD, and ME wrote the manuscript. BG, AW, PC, JL, VC, VM-S, BF, CD, SG, and ME participated in the scientific revisions of the manuscript. PAC supervised the study.

## Conflict of Interest

The authors declare that the research was conducted in the absence of any commercial or financial relationships that could be construed as a potential conflict of interest.

## Abbreviations

B[a]P: benzo[a]pyrene
AhR: aryl hydrocarbon receptor
XRE: xenobiotic response element
BPA: bisphenol-A
EDC: endocrine-disrupting compound
ERα: estrogen receptor alpha
ERβ: estrogen receptor beta
GPR30: G protein-coupled receptor 30
PXR: pregnane X receptor
PR: progesterone receptor
AIG: anchorage-independent growth
MFE: mammosphere formation efficiency
RT-qPCR: real-time quantitative polymerase chain reaction
TCDD, 2, 3, 7: 8-Tetrachlorodibenzo-p-dioxin
OS: overall survival.
