# Supplementary material for: Long-Term Exposure of Early-Transformed Human Mammary Cells to Low Doses of Benzo[a]pyrene and/or Bisphenol A Enhances Their Cancerous Phenotype via an AhR/GPR30 Interplay
Source: Front Oncol. 2020 May 29;10:712. doi: 10.3389/fonc.2020.00712 (PMC7326103; doi:10.3389/fonc.2020.00712)
Supplement: Supplementary file 1 [file Data_Sheet_1.pdf]

Supplementary Figure 1 - Characterization of the MCF10AT1 cells.

(A) Left, average number of colonies formed by MCF10AT1 and MCF10CA1a.cl1 cells in soft agar colony formation assays. Right, top, representative images of colonies; bottom, average size of the colonies. (B) Average number of mammospheres formed by MCF10AT1 and MCF10CA1a.cl1 cells. The proportion of mammosphere-forming cells isolated from the primary mammospheres greatly increased in the second generation, indicating their ability to self-renew. (C) Average size of the colonies in soft agar after 60 days of chronic exposure to B[a]P and/or BPA (10^{-10} M) of the MCF10AT1 cells. Unexposed MCF10CA1a.cl1 cells were used as a control. (D) Average size of the colonies in soft agar in the $MCF10AT1_{60d+30d}$ compared to the $MCF10AT1_{60d}$ cells. (*a.u.*, arbitrary units). All data illustrated in Supplementary Figures 1A-D represent mean ± SD of at least 3 independent experiments, in triplicate. ***$p < 0.001$, **$p < 0.01$, *$p < 0.05$ or NS (not significant) in Student t-test.

Supplementary Figure 2- BPA and/or B[a]P 10^{-10} M chronic exposure does not affect the proliferation of $MCF10AT1_{60d}$ cells.

Supplementary Figure 3- RT-qPCR analysis of *ERα, ERβ* and *PXR* mRNA expression levels represented in arbitrary units (*a.u.)* in MCF10AT1 cells. ER-positive MCF-7 and PXR-positive HG5LN PXR cells were used as controls. Values represent mean ± SD of 3 independent experiments conducted in triplicate.

Supplementary Figure 4 - Cell viability assays of MCF10AT1 cells

MCF10AT1 cells were exposed or not to (A) the AhR antagonist, GNF351, (B) the GPR30 antagonist, G15, (C) the AhR agonist, ITE, (D) and the GPR30 agonist G1. Values represent mean ± SD of 3 independent experiments conducted in triplicate. (E,F) Cell viability of

MCF10AT1 cells transfected with siRNA-AhR (E), siRNA-GPR30 (F) or their respective scrambled controls. Values represent mean ± SD of at least 2 independent experiments performed in quadruplicate.

Supplementary Figure 5 – AhR, GPR30, p42/44 MAPK protein expression levels in MCF10AT1$_{60d}$ cells

Representative western blot analyses from at least 2 independent experiments: (A) AhR and GPR30 protein expression in unexposed or exposed MCF10AT1$_{60d}$ cells; (B) phospho-p42/44 MAPK and total p42/44 MAPK in unexposed or exposed MCF10AT1$_{60d}$ cells. MCF10Ca1.cl1$_{60d}$ cells were used as controls.

Supplementary Figure 1

A

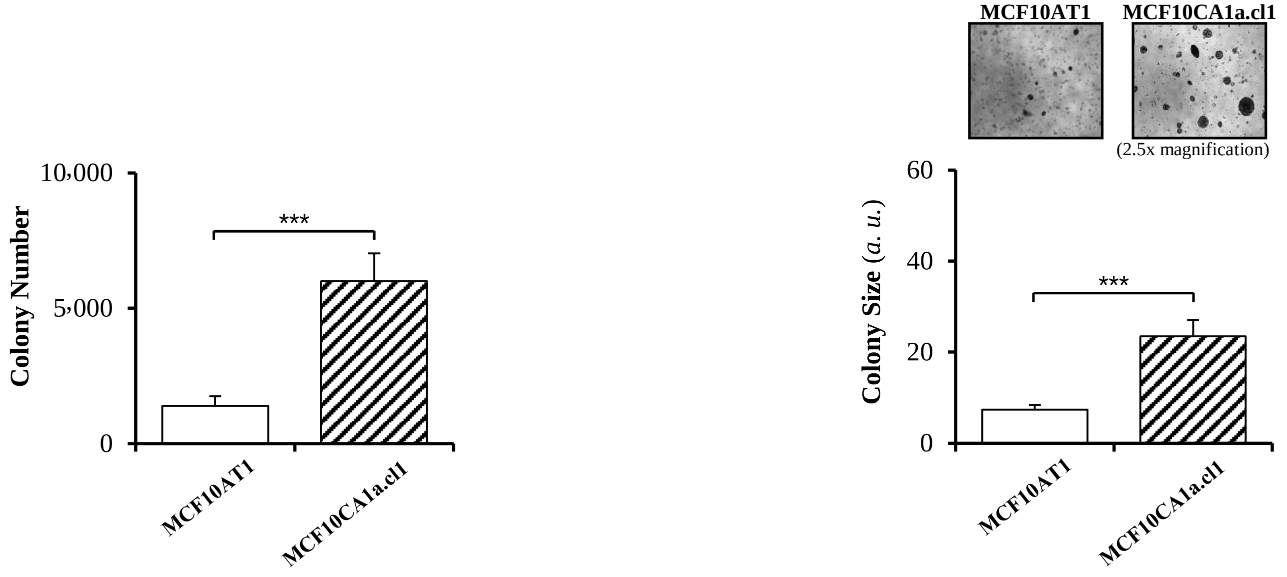

B

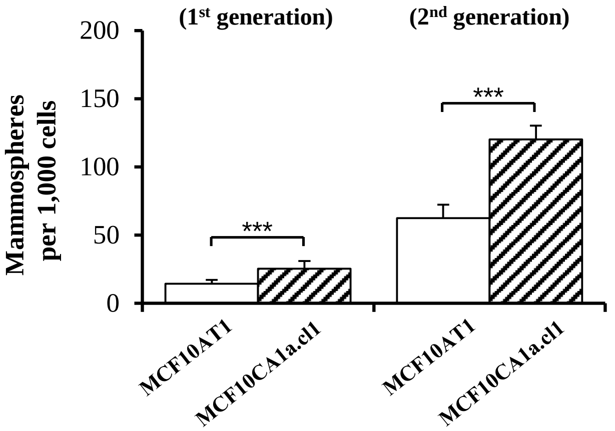

C

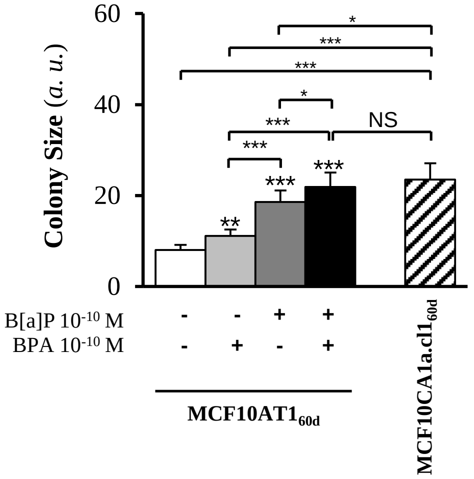

D

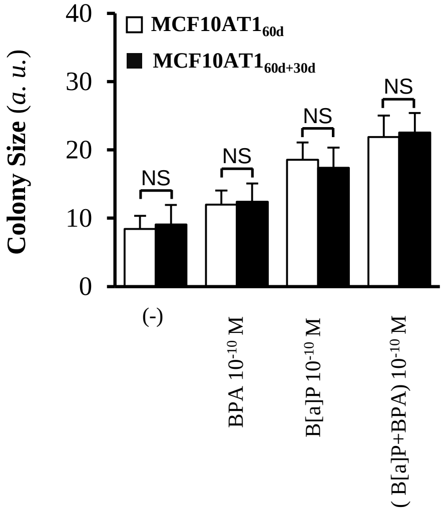

Supplementary Figure 2

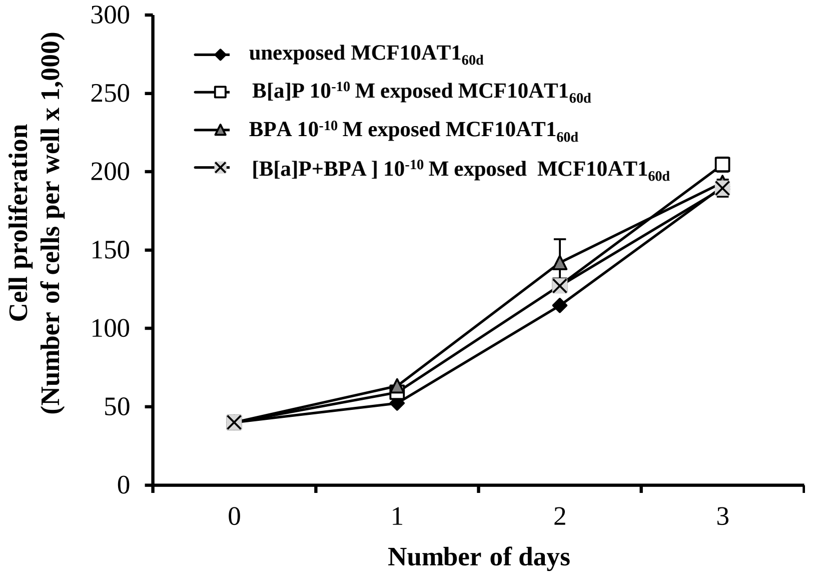

Supplementary Figure 3

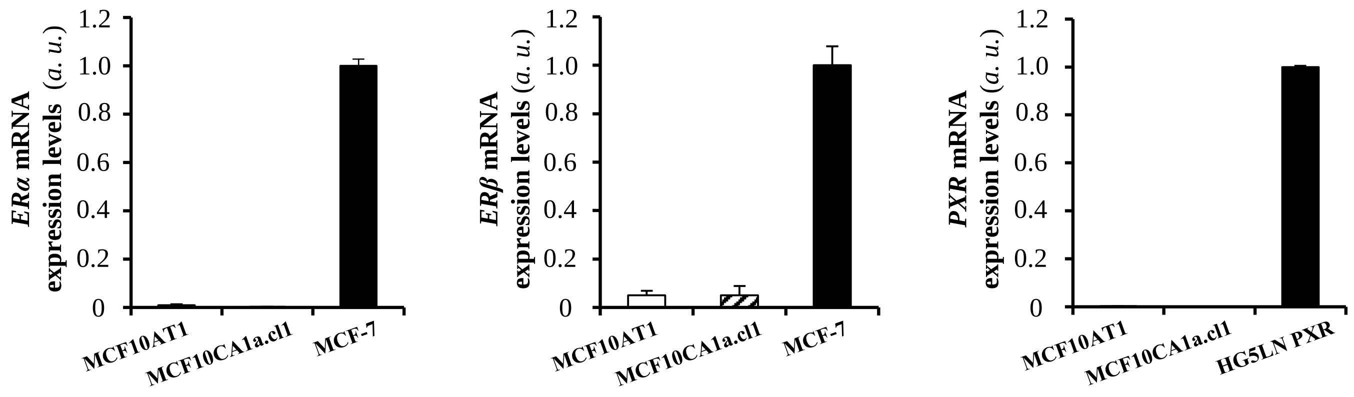

Supplementary Figure 4

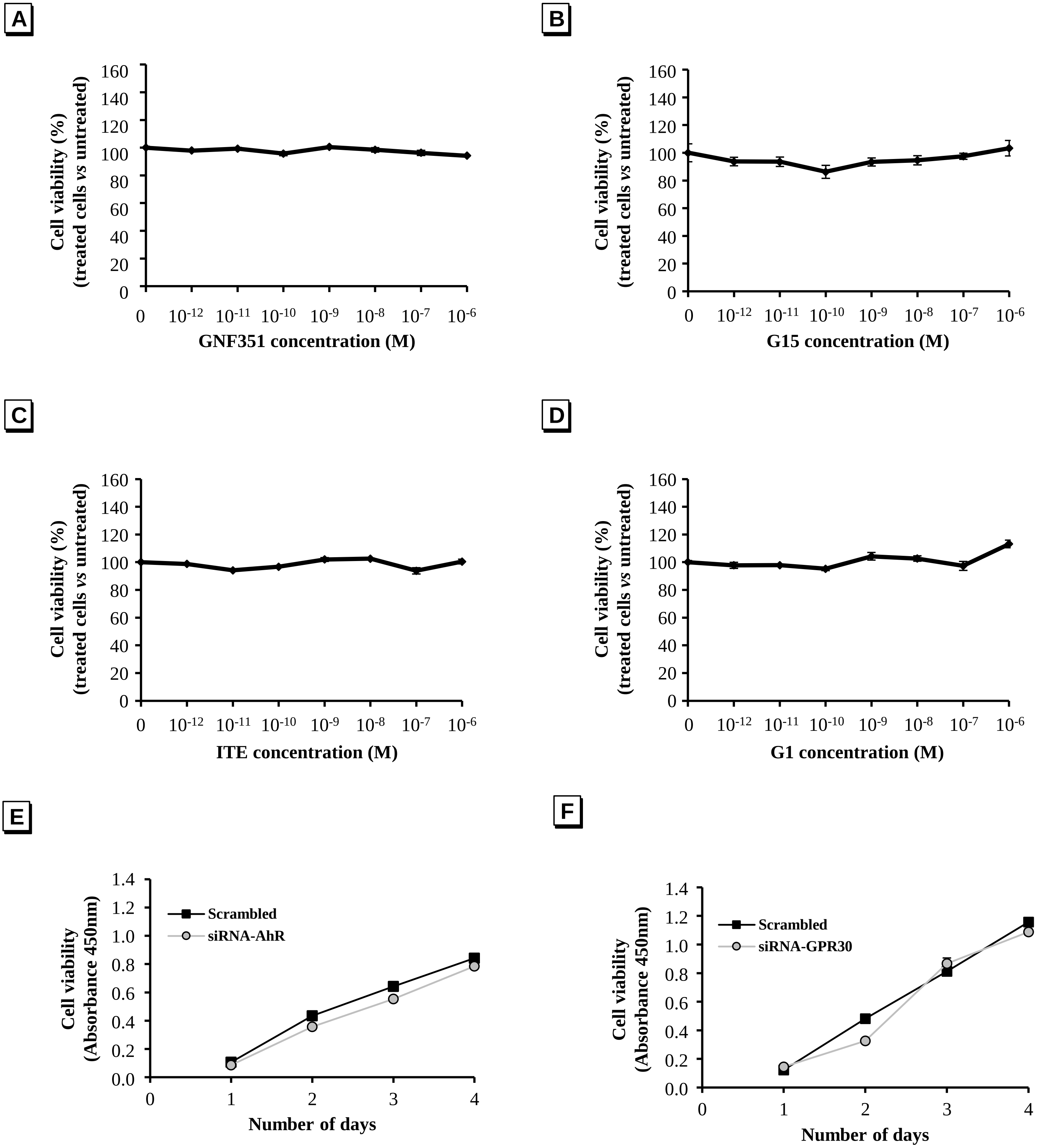

Supplementary Figure 5

A

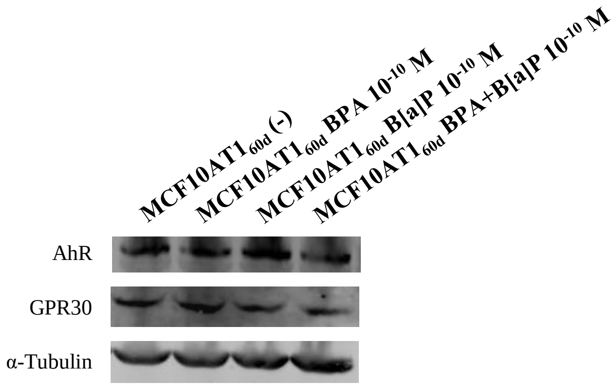

B

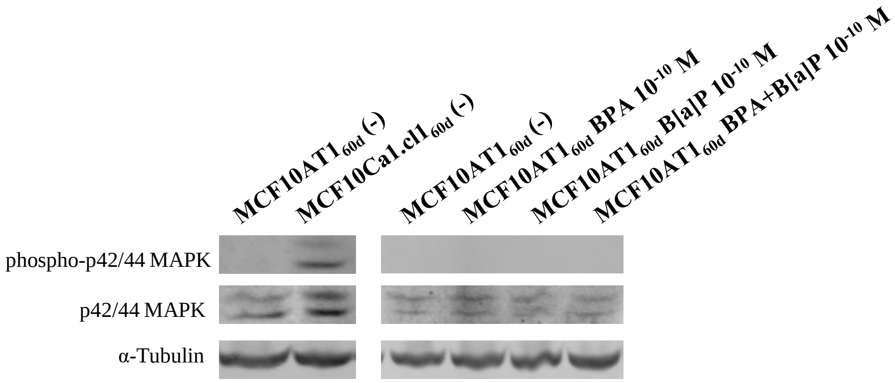

Supplementary Table 1. Primers used for RT-qPCR measurements

Gene	Forward	Reverse
ESR1 (ERα)	5’ TGTTCCAAACCCATCGTCAGT 3’	5’ CTCTATAACCAATGACCTCTCTGTGAA 3’
ESR2 (ERβ)	5’ AGAGTCCCTGGTGTGAAGCAAG 3’	5’ GACAGCGCAGAAGTGAGCATC3’
NR1I2 (PXR)	5’ GGCATGAAGAAGGAGATGAT 3’	5’ TGGGAGAAGGTAGTGTCAAA3’
AHR	5’ ACATCACCTACGCCAGTCGC 3’	5’ TCTATGCCGCTTGGAAGGAT 3’
GPER1 (GPR30)	5’ AGGGACAAGCTGAGGCTGTA 3’	5’ GTCTACACGGCACTGCTGAA 3’
CYP1A1	5’ CAGATCAACCATGACCAGAAGC 3’	5’ TTCTCACTTAACACCTTGTCGATAGC 3’
28S	5’ CGATCCATCATCCGCAATG 3’	5’ AGCCAAGCTCAGCGCAAC 3’

Supplementary Table 2. Characteristics of the 113 patients with primary breast cancers available from the Biological Resources Centre of the Leon Berard Cancer Centre (CLB cohort).

		**Number of patients**	**%**
Age (years)			
	≤ 70	96	85.0
	> 70	17	15.0
SBR grade[a,b]			
	I + II	47	42.0
	III	65	58.0
Lymph node status			
	Node negative	19	16.8
	Node positive	94	83.2
Macroscopic tumor size[b]			
	< 30 mm	43	38.4
	≥ 30 mm	69	61.6
Estrogen Receptor status			
	Positive	68	60.2
	Negative	45	39.8
Progesterone Receptor status			
	Positive	70	61.9
	Negative	43	38.1
HER2 status[b,d]			
	Positive	30	26.8
	Negative	82	73.2

[a] Scarff-Bloom-Richardson classification.
[b] Information available for 112 patients.
[c] Measured by immunohistochemistry (IHC).
[d] Measured by IHC. Few samples were validated by fluorescence *in situ* hybridization (FISH).
